# Mechanisms of low back pain: a guide for diagnosis and therapy

**DOI:** 10.12688/f1000research.8105.2

**Published:** 2016-10-11

**Authors:** Massimo Allegri, Silvana Montella, Fabiana Salici, Adriana Valente, Maurizio Marchesini, Christian Compagnone, Marco Baciarello, Maria Elena Manferdini, Guido Fanelli

**Affiliations:** 1 Department of Surgical Sciences, University of Parma, Parma, Italy; 2 Anaesthesia, Intensive Care and Pain Therapy Service, Azienda Ospedaliera Universitaria Parma Hospital, Parma, Italy

**Keywords:** low back pain, CLBP, back, spine

## Abstract

Chronic low back pain (CLBP) is a chronic pain syndrome in the lower back region, lasting for at least 3 months. CLBP represents the second leading cause of disability worldwide being a major welfare and economic problem. The prevalence of CLBP in adults has increased more than 100% in the last decade and continues to increase dramatically in the aging population, affecting both men and women in all ethnic groups, with a significant impact on functional capacity and occupational activities. It can also be influenced by psychological factors, such as stress, depression and/or anxiety. Given this complexity, the diagnostic evaluation of patients with CLBP can be very challenging and requires complex clinical decision-making. Answering the question “what is the pain generator” among the several structures potentially involved in CLBP is a key factor in the management of these patients, since a mis-diagnosis can generate therapeutical mistakes. Traditionally, the notion that the etiology of 80% to 90% of LBP cases is unknown has been mistaken perpetuated across decades. In most cases, low back pain can be attributed to specific pain generator, with its own characteristics and with different therapeutical opportunity. Here we discuss about radicular pain, facet Joint pain, sacro-iliac pain, pain related to lumbar stenosis, discogenic pain. Our article aims to offer to the clinicians a simple guidance to identify pain generators in a safer and faster way, relying a correct diagnosis and further therapeutical approach.

## Introduction

Low back pain (LBP) is the most common musculoskeletal condition affecting the adult population, with a prevalence of up to 84% ^1^. Chronic LBP (CLBP) is a chronic pain syndrome in the lower back region, lasting for at least 12 weeks ^2^. Many authors suggest defining chronic pain as pain that lasts beyond the expected period of healing, avoiding this close time criterion. This definition is very important, as it underlines the concept that CLBP has well-defined underlying pathological causes and that it is a disease, not a symptom. CLBP represents the leading cause of disability worldwide and is a major welfare and economic problem ^1^. Given this complexity, the diagnostic evaluation of patients with LBP can be very challenging and requires complex clinical decision-making. Answering the question, “what is the pain generator?” among the several structures potentially involved in CLBP is a key factor in the management of these patients, since a diagnosis not based on specific pain generator can lead to therapeutic mistakes. This article aims to provide a brief clinical guide that could help in the identification of pain generators through a careful anatomical description, thereby directing clinicians towards the correct diagnosis and therapeutic approach.

## Low back pain epidemiology

LBP represents a major social and economic problem. The prevalence of CLBP is estimated to range from 15 to 45% in French healthcare workers ^3^; the point prevalence of CLBP in US adults aged 20–69 years old was 13.1% ^4^. The general population prevalence of CLBP is estimated to be 5.91% in Italy ^5^. The prevalence of acute and CLBP in adults doubled in the last decade and continues to increase dramatically in the aging population, affecting both men and women in all ethnic groups ^6^. LBP has a significant impact on functional capacity, as pain restricts occupational activities and is a major cause of absenteeism ^7– 9^. Its economic burden is represented directly by the high costs of health care spending and indirectly by decreased productivity ^7, 9^. These costs are expected to rise even more in the next few years. According to a 2006 review, the total costs associated with LBP in the United States exceed $100 billion per year, two-thirds of which are a result of lost wages and reduced productivity ^10^.

## Looking for the pain generator

LBP symptoms can derive from many potential anatomic sources, such as nerve roots, muscle, fascial structures, bones, joints, intervertebral discs (IVDs), and organs within the abdominal cavity. Moreover, symptoms can also spawn from aberrant neurological pain processing causing neuropathic LBP ^11, 12^. The diagnostic evaluation of patients with LBP can be very challenging and requires complex clinical decision-making. Nevertheless, the identification of the source of the pain is of fundamental importance in determining the therapeutic approach ^13^. Furthermore, during the clinical evaluation, a clinician has to consider that LBP can also be influenced by psychological factors, such as stress, depression, and/or anxiety ^14, 15^. History should also include substance use exposure, detailed health history, work, habits, and psychosocial factors ^16^. Clinical information is the leading element that drives the initial impression, while magnetic resonance imaging (MRI) should be considered only in the presence of clinical elements that are not definitely clear or in the presence of neurological deficits or other medical conditions ^17^. The recommendation of the American College of Radiology is not to do imaging for LBP within the first 6 weeks unless *red flags* are present. They include recent substantial trauma or milder trauma in those over 50 years old, weight loss or fever with no known cause, immunosuppression, a previous cancer diagnosis, intravenous drug use, sustained corticosteroids use or osteoporosis, being over 70 years old, and focal neurologic deficit with progressive or disabling symptoms ^18– 20^.

Imaging findings are weakly related to symptoms. In one cross-sectional study of asymptomatic persons aged 60 years or older, 36% had a herniated disc, 21% had spinal stenosis, and more than 90% had a degenerated or bulging disc ^21^.

Although it is not possible to gauge accurately, it is easy to believe that these conditions could have a yearly cost, directly and indirectly, of more than $50 billion and conceivably up to $100 billion ^22^. A recent study estimated that lumbar radiography was performed 66 million times in the United States in 2004, with a cost of $54 for each exam ^23^. Although estimates vary substantially depending on geographic location, insurance status, and other factors, costs of MRI seem to be 10 to 15 times higher ^23, 24^.

The most recent guidelines for clinicians suggest that when faced with LBP patients, the clinician should go through a careful diagnosis of the mechanisms that sustain acute and/or chronic pain. Treatment has to be addressed specifically to these mechanisms. In this manner, we could avoid the common mistake of making the diagnosis of “simply low back pain”, resulting in improper treatment of a definition and not a complex disease. As chronic LBP could have simultaneous multiple pain generators, a multi-disciplinary diagnosis and multimodal treatment is necessary ^25^.

## Anatomy of the low back

The lumbar spine consists of five vertebrae (L1–L5). The complex anatomy of the lumbar spine is a combination of these strong vertebrae, linked by joint capsules, ligaments, tendons, and muscles, with extensive innervation. The spine is designed to be strong, since it has to protect the spinal cord and spinal nerve roots. At the same time, it is highly flexible, providing for mobility in many different planes.

The mobility of the vertebral column is provided by the symphyseal joints between the vertebral bodies, with an IVD in between. The facet joints are located between and behind adjacent vertebrae, contributing to spine stability. They are found at every spinal level and provide about 20% of the torsional (twisting) stability in the neck and low back segments ^26^. Ligaments aid in joint stability during rest and movement, preventing injury from hyperextension and hyperflexion. The three main ligaments are the anterior longitudinal ligament (ALL), posterior longitudinal ligament (PLL), and ligamentum flavum (LF). The canal is bordered by vertebral bodies and discs anteriorly and by laminae and LF posteriorly. Both the ALL and PLL run the entire length of the spine, anteriorly and posteriorly, respectively. Laterally, spinal nerves and vessels come out from the intervertebral foramen. Beneath each lumbar vertebra, there is the corresponding foramen, from which spinal nerve roots exit. For example, the L1 neural foramina are located just below the L1 vertebra, from where the L1 nerve root exits.

IVDs are located between vertebrae. They are compressible structures able to distribute compressive loads through osmotic pressurization. In the IVD, the annulus fibrosus (AF), a concentric ring structure of organized lamellar collagen, surrounds the proteoglycan-rich inner nucleus pulposus (NP). Discs are avascular in adulthood, except for the periphery. At birth, the human disc has some vascular supply but these vessels soon recede, leaving the disc with little direct blood supply in the healthy adult ^27^. Hence, metabolic support of much of the IVD is dependent on the cartilaginous endplates adjacent to the vertebral body. A meningeal branch of the spinal nerve, better known as the recurrent sinuvertebral nerve, innervates the area around the disc space ^28^.

The lumbar spine is governed by four functional groups of muscles, split into extensors, flexors, lateral flexors, and rotators. The lumbar vertebrae are vascularized by lumbar arteries that originate in the aorta. Spinal branches of the lumbar arteries enter the intervertebral foramen at each level, dividing themselves into smaller anterior and posterior branches ^29^. The venous drainage parallels the arterial supply ^30^.

Typically, the end of the spinal cord forms the conus medullaris within the lumbar spinal canal at the lower margin of the L2 vertebra ^31^. All lumbar spinal nerve roots stem from the connection between the dorsal or posterior (somatic sensory) root from the posterolateral aspect of the spinal cord and the ventral or anterior (somatic motor) root from the anterolateral aspect of the cord ^31^. The roots then flow down through the spinal canal, developing into the cauda equina, before exiting as a single pair of spinal nerves at their respective intervertebral foramina. Cell bodies of the motor nerve fibers can be found in the ventral or anterior horns of the spinal cord, whereas those of the sensory nerve fibers are in the dorsal root ganglion (DRG) at each level. One or more recurrent meningeal branches, known as the sinuvertebral nerves, run out from the lumbar spinal nerves. The sinuvertebral nerve, or Luschka’s nerve, is a recurrent branch created from the merging of the grey ramus communicans (GRC) with a small branch coming from the proximal end of the anterior primary ramus of the spinal nerve. This polisegmentary mixed nerve directly re-enters the spinal canal and gives off ascending and descending anastomosing branches comprising both somatic and autonomic fibers for the posterolateral annulus, the posterior vertebral body and the periostium, and the ventral meninges ^32, 33^. The sinuvertebral nerves connect with branches from radicular levels both above and below the point of entry, in addition to the contralateral side, meaning that localizing pain from involvement of these nerves is challenging ^34^. Also, the facet joints receive two-level innervation comprising somatic and autonomic components. The former convey a well-defined local pain, while the autonomic afferents transmit referred pain.

## Pathophysiology of spinal pain

Pain is mediated by nociceptors, specialized peripheral sensory neurons that alert us to potentially damaging stimuli at the skin by transducing these stimuli into electrical signals that are relayed to higher brain centers ^35^. Nociceptors are pseudo-unipolar primary somatosensory neurons with their neuronal body located in the DRG. They are bifurcate axons: the peripheral branch innervates the skin and the central branches synapse on second-order neurons in the dorsal horn of the spinal cord ^36^. The second-order neurons project to the mesencephalon and thalamus, which in turn connect to somatosensory and anterior cingulate cortices in order to guide sensory-discriminative and affective-cognitive features of pain, respectively ^37^. The spinal dorsal horn is a major site of integration of somatosensory information and is composed of several interneuron populations forming descending inhibitory and facilitatory pathways, able to modulate the transmission of nociceptive signals ^38^. If the noxious stimulus persists, processes of peripheral and central sensitization can occur, converting pain from acute to chronic. Central sensitization is characterized by the increase in the excitability of neurons within the central nervous system, so that normal inputs begin to produce abnormal responses ^37^. It is responsible for tactile allodynia, that is pain evoked by light brushing of the skin, and for the spread of pain hypersensitivity beyond an area of tissue damage. Central sensitization occurs in a number of chronic pain disorders, such as temporomandibular disorders, LBP, osteoarthritis, fibromyalgia, headache, and lateral epicondylalgia ^39^. Despite improved knowledge of the processes leading to central sensitization, it is still difficult to treat ^40, 41^. Peripheral and central sensitization have a key role in LBP chronification. In fact, minimal changes in posture could easily drive long-lasting inflammation in the joints, ligaments, and muscles involved in the stability of the low back column, contributing to both peripheral and central sensitization. Furthermore, joints, discs, and bone are richly innervated by A delta fibers whose continuous stimulation could easily contribute to central sensitization.

## Type of spinal pain according to pain generator

In spite of the hard work done by the International Association for the Study of Pain ^41^, there remains a degree of confusion in the medical community regarding the definitions of back pain, referred pain, radicular pain, and radiculopathy. Nevertheless, a precise diagnostic assessment is necessary to indicate the right treatment. An incorrect diagnosis and use of a therapy that is not appropriate could also be related to insufficient diagnostic skills of a non physician specialized in this syndrome, attributed to a clinical and/or instrumental analysis of insufficient depth, or a therapeuthic approach geared towards controlling the symptom (pain) rather than the pain generator mechanisms ^25^.

Mostly, LBP is considered to be nonspecific ^42^, and the mistaken idea that the cause of 80 to 90% of LBP cases is unknown has persisted for decades ^43– 47^.

Muscle tension and spasm are among the most common reasons for LBP, for example, in patients with fibromyalgia. In other cases, LBP can be attributed to different pain generators, with specific characteristics, such as radicular, facet joint, sacro-iliac, and discogenic pain, as well as spinal stenosis.

### Radicular pain

Radicular pain is pain evoked by ectopic discharges emanating from an inflamed or lesioned dorsal root or its ganglion; generally, the pain radiates from the back and buttock into the leg in a dermatomal distribution ^46^. Disc herniation is the most common cause, and inflammation of the affected nerve rather than its compression is the most common pathophysiological process. Radicular pain is pain irradiated along the nerve root without neurological impairment. Even though it is nociceptive pain, it is distinguished from usual nociception because in radicular pain the axons are not stimulated along their course or in their peripheral terminals but from the perinevrium ^42, 48^. Radicular pain differs from radiculopathy in several aspects. Radiculopathy impairs conduction down a spinal nerve or its roots. The impairment of sensory fibers causes numbness (dermatomally distributed); however, blockade of motor fibers causes weakness (myotomal). Sensory or motor block may result in diminished reflexes ^46^. Although radiculopathy and radicular pain often accompany one another, radiculopathy has been observed in the absence of pain, and radicular pain may happen in the absence of radiculopathy ^48, 49^. It is important to underline that, contrary to popular belief, it is not possible to make a distinction among the patterns of L4, L5, and S1 radicular pain ^50, 51^. In fact, only when radiculopathy is seen together with radicular pain can segments be estimated. In such cases, the dermatomal distribution of numbness indicates the segment of origin rather than the distribution of pain. Lumbar disc herniation with radiculopathy can be diagnosed during clinical examination using manual muscle testing, supine straight leg raise, Lasègue sign, and crossed Lasègue sign.

If a patient’s history and physical examination findings indicate lumbar disc herniation with radiculopathy, the most suitable noninvasive test to confirm this could be an MRI. This is particularly important if it is necessary to proceed with an invasive treatment or to better define the neurological impairment. The next most appropriate test to evaluate the presence of lumbar disc herniation is computed tomography (CT) or CT myelography, which would be suitable for those individuals unable to have an MRI because it is contraindicated or those for whom MRI is inconclusive. Also, diagnosis of nerve root compression may be achieved by electrodiagnostic studies, although they are not able to distinguish between lumbar disc herniation and other causes of nerve root compression. Unfortunately, we have to remark that radiculopathy could be present without radicular pain and vice versa. For these reasons, electrodiagnostic tests are not recommended as a first-line approach but only as a second-line one in order to define if there is a concomitant presence of peripheral neuropathy or neuralgia or to follow up the impairment of the lesioned nerve ^52^.

### Facet joint syndrome

The lumbar zygapophyseal joints are the posterior articular process of the lumbar column. They are formed from the inferior process of upper vertebra and the superior articular process of lower vertebra ^53^. They are supplied by the medial branches of the dorsal rami (MBN). These joints have a large amount of free and encapsulated nerve endings ^54^ that activate nociceptive afferents and that are also modulated by sympathetic efferent fibers ^55^. Lumbar zygapophyseal or “facet” joint pain has been estimated to account for up to 30% of CLBP cases ^56^, with nociception originating in the synovial membrane, hyaline cartilage, bone, or fibrous capsule of the facet joint ^57^.

Diagnosis of facet joint syndrome is often difficult and requires a careful clinical assessment and an accurate analysis of radiological exams. Patients usually complain of LBP with or without somatic referral to the legs terminating above the knee, often radiating to the thigh or to the groin. There is no radicular pattern. Back pain tends to be off-center and the pain intensity is worse than the leg pain; pain increases with hyperextension, rotation, lateral bending, and walking uphill. It is exacerbated when waking up from bed or trying to stand after prolonged sitting. Finally, patients often complain of back stiffness, which is typically more evident in the morning ^58, 59^. Jackson was able to correlate seven features with facet pain: older age, previous episodes of LBP, normal gait, maximal pain with lumbar extension but a failure to aggravate pain with the Valsalva maneuver, and a lack of leg pain or muscle spasm ^59, 60^.

It is difficult to diagnose lumbar facet syndrome using radiology as there are no pathognomonic findings to look for 61. With MRI, we can find non-specific signs of arthrosis, osteophytes, and hypertrophy of flaval ligaments. However, if we want to better study arthrosis problems, CT is the preferred imaging method, even if radiation exposure should be kept in mind ^58^. One of the most important exams is provided by X-rays, especially dynamic projections, that can show column instability (listhesis that could be increased with flexion and extension of the low back column) with a clear overload of these joints ^60^. In conclusion, despite the contribution from neuroimaging, history and clinical examination remain fundamental steps in the diagnosis of facet joint syndromes.

### Sacroiliac joint pain

Sacroiliac joints (SIJs) are dedicated to providing stable but flexible support for the upper body ^62, 63^. SIJs are involved in sacral movement, which additionally directly influences the discs and almost certainly the higher lumbar joints. Its innervation is still not well known but has been reported to be by branches from the ventral lumbopelvic rami ^64^; however, this has not yet been confirmed. On the other hand, several authors have reported innervation of the SIJ by small branches from the posterior rami ^65, 66^. In a 2012 study by Patel et al. ^66^, the authors demonstrated that SIJ pain was successfully attenuated using neurotomy of the L5 dorsal primary ramus and lateral branches of the dorsal sacral rami from S1 to S3 ^63^. Hence, there is sufficient evidence that this procedure has an important value for establishing diagnosis and prognosis. The SIJ is well recognized as a source of pain in many patients who present with CLBP ^67, 68^. It is thought that pain could be generated by ligamentous or capsular tension, extraneous compression or shear forces, hypermobility orhypomobility, altered joint mechanics, and myofascial or kinetic chain dysfunction causing inflammation ^69^. Intra-articular sources of SIJ pain include osteoarthritis; extra-articular sources include enthesis/ligamentous sprain and primary enthesopathy. In addition, ligamentous, tendinous, or fascial attachment and other cumulative soft tissue injuries that may occur posterior to the dorsal aspect of the SIJ may be a source of discomfort. In physical examination, it is important to examine the movement of the joint, for example with a stress test, consisting of pressing down on the iliac crest (pelvis) or upper thigh, which may reproduce the patient’s pain.

SIJ pain is often underdiagnosed. It has to be considered in every situation in which the patient complains of postural LBP that worsens in a sitting position and with postural changes. Furthermore, it is possible that SIJ pain is often strictly related to facet joint syndromes as both are related to postural problems.

Finally, it is important to consider that SIJ pain could also be a sign of rheumatic disease. MRI findings of articular effusion and inflammation (especially if bilateral) can alert the clinician to consider this condition.

### Lumbar spinal stenosis

Lumbar spinal stenosis (LSS) can be congenital ^70^ or acquired (or both). It could be determined by inflammatory/scar tissue after spine surgery or, even in absence of previous surgery, by disc herniation, thickening of the ligaments, or hypertrophy of the articular processes ^71^. The majority of cases of LSS are degenerative, related to changes in the spine with aging ^72^. LSS is determined by a progressive narrowing of the central spinal canal and the lateral recesses and consequent compression of neurovascular structures ^73^. Usually, the diameter of the normal lumbar spinal canal varies from 15 to 27 mm. We can define lumbar stenosis as a spinal canal diameter of less than 10 mm, even though a stenosis with diameter of 12 mm or less in some patients can be symptomatic. The normal foraminal height varies from 20 to 23 mm, with the indicator of potential foraminal stenosis as 15 mm or less ^74^. Degenerative LSS is the most common indication for spinal surgery in people older than 65 years of age ^73^. The most frequent symptoms of lumbar stenosis are midline back pain, radiculopathy with neurologic claudication, motor weakness, paresthesia, and impairment of sensory nerves ^75^. Symptoms may have a different distribution depending on the type of LSS. If the LSS is central, there may be involvement of the area between the facet joints, and pain may be bilateral in a non-dermatomal distribution. With lateral recess stenosis, symptoms are usually found dermatomally because specific nerves are compressed, resembling unilateral radiculopathy ^76^. Trunk flexion, sitting, stooping, or lying can all ease the discomfort, while prolonged standing or lumbar extension can aggravate the pain. Sitting or lying down become less effective in alleviating pain as the condition progresses, and rest pain or a neurogenic bladder can develop in severe cases ^76, 77^. Neurogenic claudication pain is the classical symptom of LSS, caused by venous congestion and hypertension around nerve roots. Pain is exacerbated by standing erect and by downhill ambulation but alleviated with lying supine more than prone, sitting, squatting, and lumbar flexion ^78, 79^.

LSS is generally diagnosed based on a combination of history, physical examination, and imaging ^75^. The most useful findings from the history are age, radiating leg pain that is exacerbated by standing up or walking, and the absence of pain when seated ^80^. The gait and posture after walking may reveal a positive “stoop test” ^79, 80^, performed by asking the patient to walk briskly. As the pain intensifies, patients may complain of sensory symptoms followed by motor symptoms, and if they assume a stooped posture, symptoms may improve ^80^. If patients sit in a chair bent forward, they may have the same relief ^81^.

The recommended method for confirming the diagnosis of LSS is MRI, which facilitates the assessment of the spinal canal and the anatomic relationship between spinal and neural elements ^80^. The natural course of untreated LSS is unclear. The North American Spine Society (NASS) clinical guidelines concluded that the natural course is favorable in a third to a half of patients with clinically mild to moderate LSS ^82^. Other reviews suggest that the condition may deteriorate in some patients and improve in about a third of others, with most patients remaining unchanged for up to 8 years of follow-up ^83– 85^.

### Discogenic pain

Disc degeneration (DD) has been estimated as the source of CLBP in 39% of cases ^86^. Its symptoms are aspecific, axial, and without radicular radiation and they occur in the absence of spinal deformity or instability. DD is often a diagnosis of exclusion among other types of CLBP. Pathologically, it is characterized by the degradation, within the disc, of the NP matrix with accompanying radial and/or concentric fissures in the AF ^87^.

Despite numerous recent advances, the main issue is how inflammation is initiated and sustained to lead to CLBP. A possible explanation could involve the growth of nerves capable of signaling pain deep into the annular structures ^88^. Another hypothesis involves a class of molecules, called damage-associated molecular patterns (DAMPs), including hyaluronic acid and fibronectin fragments, able to stimulate sterile inflammation of the disc through the action of pro-inflammatory cytokines (IL-1beta, IL-6, and IL-8) and matrix degrading enzymes (MMP-1, MMP-3, and MMP-13) ^87^. Also, subclinical anaerobic bacterial infection, encouraged by hypoxic conditions, could have a role in the development of discogenic pain ^88^.

Imaging MRI can detect changes in the endplates and in the vertebral bone marrow, such as edema in the vertebral bodies (Modic type 1). Clinical trials have demonstrated that some patients suffering from LBP have improvement following amoxicillin-clavulanate ^88, 89^. Moreover, diabetes increases the risk of developing painful DD because advanced glycation end products (AGEs) induce catabolism and promote inflammation ^90^.

MRI cannot definitively demonstrate whether a disc is painful ^91^. Provocation discography aims at reproducing patients’ pain through contrast injection during live fluoroscopy plus CT imaging for clarifying associated morphological abnormalities of the disc ^92^. The clinical utility of discography and its diagnostic accuracy is, however, a matter of controversy because of poor specificity. Beyond the reported complications as discitis, neurologic injury, visceral injury, and dye reactions ^93^, it’s been demonstrated that the needle puncture of the lumbar disc may lead to accelerated MRI-documented DD. The mechanism is likely multifactorial: structural damage caused by the needle, pressurization, and toxicity of the contrast media ^94^.

**Figure 1.**
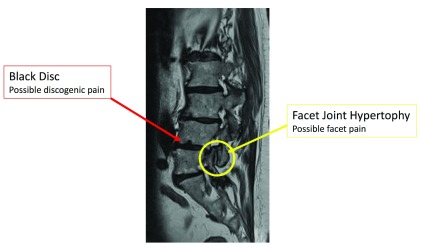
MRI sagittal image showing an abnormal alignment of lumbar vertebrae; black discs (red arrow) are pathogenetic for discogenic pain; facet joint hypertrophy (yellow arrow) is pathogenetic for facet joint pain.

**Figure 2.**
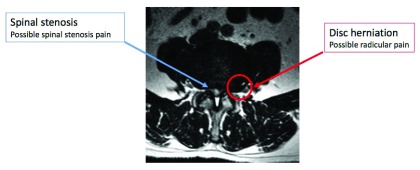
MRI axial image showing reduction in the size of the spinal canal (blue arrow), a pathogenetic finding in spinal stenosis; the red arrow shows radicular compression that can cause radicular pain.

## Concluding remarks

LBP is one of the most common symptoms and conditions motivating individuals to seek medical consultation. The effects of back pain on society are significant, both epidemiologically and economically, and this is likely to only further increase owing to a combination of shifting attitudes and expectations, medical management techniques, and social provision.

Hence, LBP must always be addressed as a complex disease in which it is mandatory that an accurate diagnosis of pain generators is determined before starting any treatment. All the guidelines currently avalaible stress the importance of a multimodal and multidisciplinary approach in order to determine a strategy to solve the problem and not simply alleviate symptomatic pain. Finally, a careful follow up is important to adapt our therapeuthic strategies to dynamic clinical manifestations of CLBP. 
